# ImmQuant: a user-friendly tool for inferring immune cell-type composition from gene-expression data

**DOI:** 10.1093/bioinformatics/btw535

**Published:** 2016-08-16

**Authors:** Amit Frishberg, Avital Brodt, Yael Steuerman, Irit Gat-Viks

**Affiliations:** Department of Cell Research and Immunology, George S. Wise Faculty of Life Sciences, Tel Aviv University, Tel Aviv 6997801, Israel

## Abstract

**Summary**: The composition of immune-cell subsets is key to the understanding of major diseases and pathologies. Computational deconvolution methods enable researchers to investigate immune cell quantities in complex tissues based on transcriptome data. Here we present ImmQuant, a software tool allowing immunologists to upload transcription profiles of multiple tissue samples, apply deconvolution methodology to predict differences in cell-type quantities between the samples, and then inspect the inferred cell-type alterations using convenient visualization tools. ImmQuant builds on the DCQ deconvolution algorithm and allows a user-friendly utilization of this method by non-bioinformatician researchers. Specifically, it enables investigation of hundreds of immune cell subsets in mouse tissues, as well as a few dozen cell types in human samples.

**Availability and implementation:** ImmQuant is available for download at http://csgi.tau.ac.il/ImmQuant/.

**Contact:** iritgv@post.tau.ac.il

**Supplementary information:** Supplementary data are available at *Bioinformatics* online.

## 1 Introduction

The repertoire of changes in immune-cell types between different physiological states and the accurate determination of these changes can facilitate biomedical research, diagnosis and treatment. Despite this important attribute, experimental quantification of cell types has remained relatively low throughput. One attractive approach is to apply deconvolution techniques to infer the composition of cell types based on gene-expression data, thus avoiding the need for experimental cell sorting (Shen-Orr and Gaujoux, 2013).

Using immune deconvolution methods, the quantities of cell subsets within a given heterogeneous tissue can be inferred based on (i) transcription profiling of a tissue sample, (ii) prior knowledge about the signatures of immune-cell subsets (referred to as ‘reference data’) and (iii) a group of effective gene markers for each of the cell subsets in the reference data. Most deconvolution methods were proven useful in human applications, where the reference datasets are of intermediate sizes [22 and 38 cell types in the IRIS and DMAP reference datasets, respectively (Abbas *et al.*, 2005; Novershtern *et al.*, 2011)]. However, these methods are typically not scalable to the substantially larger number of reference signatures that are available in mouse [207 cell types in the ImmGen dataset (Heng and Painter, 2008)].

To address this, we recently introduced the digital cell quantifier (DCQ) deconvolution algorithm, which can robustly infer differences between murine samples at a scale of a few hundreds of cell types (Altboum *et al.*, 2014). DCQ is focused on fold changes in cell-type quantities between samples while assuming sparse changes in cell-type composition (that is, assuming that only a subset of the cell types differs between the compared samples). Using this assumption, DCQ can be effectively applied on mouse tissues utilizing the cell types contained in the ImmGen dataset. Indeed, the accuracy of DCQ on mouse samples was validated using several simulations and experimental approaches (e.g., Frishberg *et al.*, 2015).

Since DCQ was so far applied only on mouse data, we decided to test the performance of DCQ in the case of human data as well. To facilitate the use of DCQ by immunologists, we developed ImmQuant, a freely available multiplatform software (running on both Windows and Mac) that enables users to apply DCQ on transcription profiling in heterogeneous samples, both human and mouse, and observe the inferred alterations in composition of immune-cell populations using convenient visualizations. Our simulations show that ImmQuant can withstand the large number of predicted cell subsets in mouse data while maintaining a good accuracy in human data. We show that DCQ can be usefully applied, for example, to examine immune cell-type alterations in murine lung infection and in human Sjögren’s syndrome pathogenesis.

## 2 Methods

**The DCQ algorithm**. ImmQuant performs deconvolution using the DCQ algorithm (Altboum *et al.*, 2014). DCQ takes as input (i) a relative expression profile, calculated as the transcription fold-change between two heterogeneous samples; (ii) a reference dataset, consisting of transcriptional signatures of immune-cell subsets and (iii) a list of informative marker genes. Using this input, DCQ decomposes the relative expression profile into cell-type differences between the two samples (Supplementary Methods for full details).

**Reference data and marker genes**. ImmQuant benefits from two reference datasets in human. First, the IRIS dataset (Abbas *et al.*, 2005), which consists of 22 transcription profiles of immune-cell types isolated from normal human blood. Second, the DMAP dataset (Novershtern *et al.*, 2011) that carries 38 different cell types, including 21 intermediate maturation states isolated from umbilical cord blood and 17 terminally differentiated cell types isolated from peripheral blood. In mouse, ImmQuant utilizes the 207 cell types in ImmGen (Heng and Painter, 2008), consisting of cell types isolated from 22 different tissues. Cell types were profiled either in the basal state or following a variety of extracellular stimuli. For each reference data, marker genes were collected as previously reported (Supplementary Methods).

## 3 Results

We first evaluated the quality of DCQ using synthetic data, focusing on assessment of performance using the low and high number of cell types in the human and mouse reference collections, respectively. Our analysis showed that DCQ accurately predicts relative cell-type quantities across a range of numbers of cell types. In comparison with existing methods, DCQ achieves the best performance when using mouse data and is the second best for human data. A detailed description of data generation and performance evaluation is provided in Supplementary Methods, Information S1 and Table S1.

**Fig. 1.**
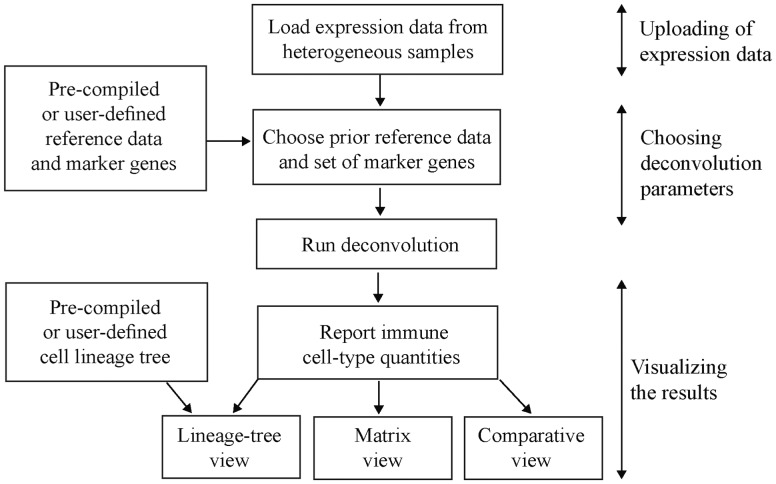
The ImmQuant pipeline

We next aimed to provide a software tool to enable non-bioinformatician users to apply DCQ on their own datasets. ImmQuant implements the entire analysis pipeline: first, import of the transcription-profiling data; next, selection of reference data and marker genes and finally, visualization of the resulting cell-type quantities (Fig. 1 and Supplementary Fig. S1). Reference data and marker genes can either be selected from a collection of pre-compiled options (two reference datasets in human and one dataset in mouse) or, alternatively, be uploaded by the user. The ability to add user-defined reference datasets opens the way to future deconvolution analyses of newly identified cell types. Finally, ImmQuant allows two types of fold-change calculations: relative to the average of all samples and relative to a selected subset of control samples. We note that ImmQuant does not support data pre-processing, and the input gene-expression is assumed to be normalized beforehand (Supplementary Methods). Deconvolution takes several minutes for a dataset of 100 samples (Supplementary Table S1).

ImmQuant provides several options for visualization of the inferred cell-type quantities, including a matrix viewer, a comparative viewer and projection of the cell-type quantities on top of the haematopoietic-lineage tree (Supplementary Fig. S1c–e). The predicted quantities and various visualizations can be easily exported for further analysis. A detailed description of the ImmQuant pipeline is provided in Supplementary Methods. Supplementary Information S2 demonstrates ImmQuant’s capabilities in two case studies: murine lung samples during influenza infection and human parotid gland tissue in Sjögren’s syndrome.

## 4 Summary

We developed ImmQuant, a software for studying the composition of immune cells in complex tissues on the basis of transcription profiles. ImmQuant was designed for non-bioinformatician users to facilitate the immunological interpretation of clinical data and disease models. ImmQuant performs well in both human and mouse data, providing the first user-friendly immune deconvolution application that can exploit the sizeable number of murine cell-type signatures.

## Supplementary Material

Supplementary Data
