# Supplementary material for: ImmQuant: a user-friendly tool for inferring immune cell-type composition from gene-expression data
Source: Bioinformatics. 2016 Aug 16;32(24):3842–3. doi: 10.1093/bioinformatics/btw535 (PMC5167062; doi:10.1093/bioinformatics/btw535)
Supplement: Supplementary Data [file supp_32_24_3842__index.html]

ImmQuant: a user-friendly tool for inferring immune cell-type composition from gene-expression data — ImmQuant: a user-friendly tool for inferring immune cell-type composition from gene-expression data — Supplementary Data 

# ImmQuant: a user-friendly tool for inferring immune cell-type composition from gene-expression data

## Supplementary Data

files

- Supplementary Data - pdf file
