# Supplementary material for: ImmQuant: a user-friendly tool for inferring immune cell-type composition from gene-expression data
Source: Bioinformatics. 2016 Aug 16;32(24):3842–3. doi: 10.1093/bioinformatics/btw535 (PMC5167062; doi:10.1093/bioinformatics/btw535)
Supplement: Supplementary Data [file supp_btw535_ImmQuant_Supplementary_data.pdf]

**Supplementary Material**

# ImmQuant: a user-friendly tool for inferring immune cell type composition from gene-expression data

**Amit Frishberg, Avital Brodt, Yael Steuerman and Irit Gat-Viks**

Department of Cell Research and Immunology, George S. Wise Faculty of Life Sciences, Tel Aviv University, Tel Aviv, Israel

- Supplementary Tables and Figures (p. 2-3)
- Supplementary Methods (p. 4 - 9)
- Supplementary Information 1 (p. 10 - 11)
- Supplementary Information 2 (p. 12 - 14)
- References (p. 15)

## Supplementary Tables and Figures

| Deconvolution Software | | ImmQuant | Cibersort[1] | I-NNLS[2] | Qprog[3] | DSA[4] |
|---|---|---|---|---|---|---|
| Software type | | Stand-alone application | Web server | R script | R script | R script |
| Organism (precompiled) | | Human, mouse | Human | Human | Human | Human |
| Running time (seconds) | Human (IRIS) | 0.2294 | 3.99 | 0.0477 | 0.0045 | 0.0067 |
| | Human (DMAP) | 0.1198 | 73.25 | 0.0047 | 0.0045 | 0.0098 |
| | Mouse (Immgen) | 0.4638 | 1040.08 | 2.2323 | 0.2437 | n.a. |
| Accuracy of prediction | Human (DMAP) | Good | Very good | Fair | Fair | Fair |
| | Mouse (Immgen) | Good | poor | Poor | Poor | n.a. |

**Supplementary Table 1. Comparison to other tools.** The table summarizes the ImmQuant software capabilities in comparison with other tools. Running time was monitored using Intel i7-3740QM 2.7GHz (16GB) machine for a single sample. Accuracy of prediction was evaluated based on our synthetic data analysis in Supplementary Information 1. Compared tools: [1]Newman, A.M., et al., 2015; [2]Abbas, A.R., et al., 2009; [3]Gong, et al., 2011; [4]Zhong, et al., 2013. DSA could not be applied in mouse data due to the large number of cell types.

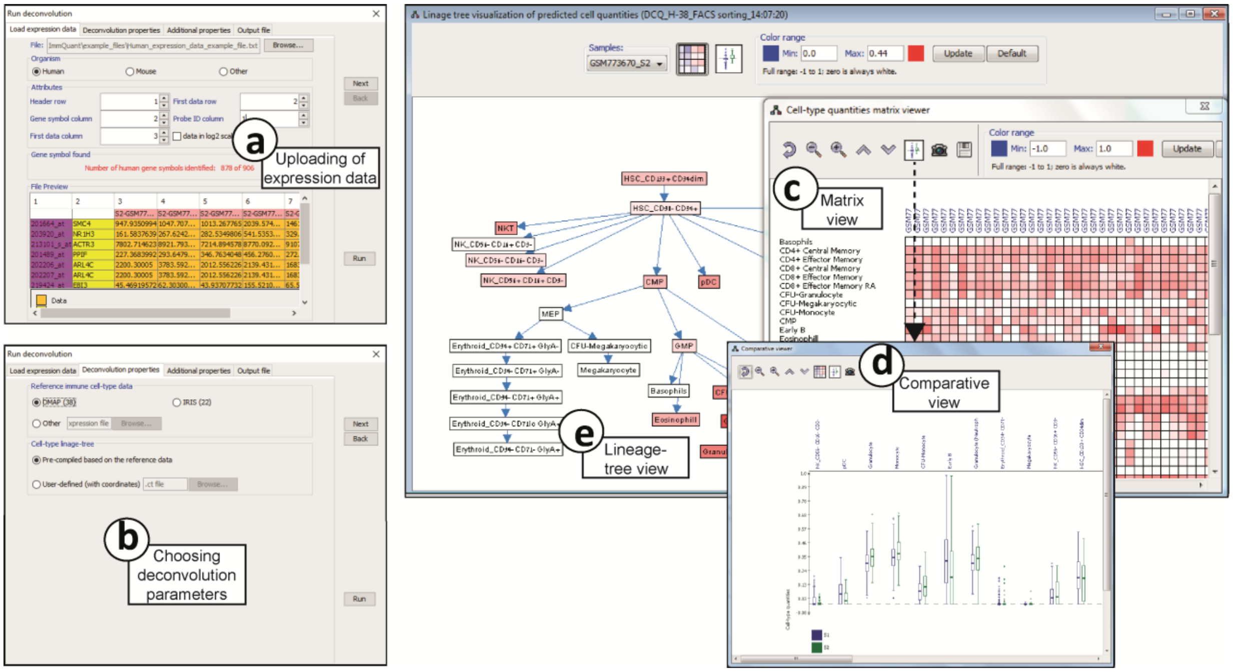


**Supplementary Figure 1**. **User interface in ImmQuant.** Shown are uploading options (**a**), deconvolution options (**b**), and output reports, including a matrix view (**c**), a comparative view (**d**) and a lineage-tree view (**e**).

# Supplementary Methods

## Supplementary Methods 1: The DCQ algorithm

Deconvolution algorithms decompose the gene expression from a given heterogeneous tissue into the abundance of individual cell types within the tissue. The objective is to find a solution to the following equation:

$$\mathbf{y} = \mathbf{X} \cdot \boldsymbol{\beta} + \mathbf{e} \quad (1)$$

- $\mathbf{y} = (y_1,..., y_m)^T$ is the dependent (response) variable, providing the measured expression level of each gene $j \in \{1,...,m\}$ in a given heterogeneous tissue.
- $\mathbf{X}$ is an $m \times n$ matrix of explanatory (regressor) variables, referred to as the *reference data*. Each entry $x_{j,c}$ corresponds to the expression of a gene $j \in \{1,...,m\}$ in cell type $c \in \{1,...,n\}$.
- $\boldsymbol{\beta}$ is a column vector of regression coefficients $(\beta_1,...,\beta_n)^T$. $\beta_c$ represents the quantity of cell-type $c$ within the heterogeneous tissue, referred to collectively as the *cell-type quantities.*
- $\mathbf{e}$ is a column $m$-dimensional vector that captures noise factors affecting $\mathbf{y}$.

In deconvolution, immune cell-type quantities ($\boldsymbol{\beta}$) are calculated on the basis of gene expression in a heterogeneous tissue and a reference dataset ($\mathbf{y}$ and $\mathbf{X}$, respectively). To attain realistic predictions and account for the complexity of the output, various deconvolution algorithms solve Eq. 1 while posing additional constraints on the inferred cell-type quantities. Such approaches include the Cibersort (Newman, et al., 2015), DSA (Zhong, et al., 2013), QProg (Gong, et al., 2011) and Iterative Non-Negative Least Squares (I-NNLS) (Abbas, et al., 2009) algorithms.

ImmQuant solves the deconvolution problem using the DCQ algorithm (Altboum, et al., 2014). Unlike the abovementioned methods, DCQ is focused on alterations in cell type quantities between two samples (test versus control samples). Using DCQ, the input data comprise the *relative* expression profile (fold-change) between two heterogeneous samples; in accordance, the output refers to the *relative* cell-type quantities, ranging between negative coefficients (decrease in quantities) and positive values (increase in quantities). Specifically, DCQ utilize the equation $\mathbf{y} = \mathbf{X} \cdot \boldsymbol{\beta} + \mathbf{e}$ where $\mathbf{y} = (y_1,..., y_m)^T$ is the *relative expression level* of each gene $j \in \{1,...,m\}$ between two heterogeneous tissues, and $\boldsymbol{\beta}$ is a column vector of regression coefficients $(\beta_1,...,\beta_n)^T$ where $\beta_c$

represents the altered quantity of cell-type *c* between the two heterogeneous tissues, referred to as the *relative cell-type quantities*; $\mathbf{X}$ and $\mathbf{e}$ are the reference data and noise factors as defined above. To account for the complexity of the output, DCQ applies the 'elastic net' regularization of the linear regression (Zou and Hastie, 2005), solved using the 'glmnet' R function.

We note that DCQ is applied in the same manner on any given organism. The only difference between organisms is the input reference dataset, which requires an accompanying selection of marker genes, as detailed below.

## Supplementary Methods 2: Selection of marker genes.

A number of studies have shown that deconvolution yields substantial improvement in the signal-to-noise ratio when the problem is solved with a relatively small set of informative genes (rather than all genes in the reference dataset). Selection of such genes—called *marker genes*—typically relies on their expression levels in the reference data. ImmQuant provides pre-compiled marker sets tailored for each reference datasets. A selection of *signature markers* was done in two steps, as described in previous publications (Abbas, et al., 2009; Newman, et al., 2015): first, utilizing a statistical t-test to select gene markers that discriminate well (with significant *P*-value) between each cell type and its two neighboring (most similar) cell types; next, among those markers, choosing the subset of markers with the highest fold change that minimizes the 'condition number' metric of the reference matrix. Overall, 2169, 1566 and 3824 genes were selected for the IRIS, DMAP and ImmGen reference datasets, respectively. We note that for the specific case of the ImmGen data, ImmQuant provides an additional set of markers, referred to as the *FACS-based marker genes set,* which was previously constructed in (Altboum, et al., 2014). Unless stated otherwise, in this study we used the signature markers.

## Supplementary Methods 3: Synthetic data analysis.

Synthetic expression data was generated as a mixture of cell types, as reported in Frishberg, et al., 2015. In brief, to generate the simulated data for a single tissue carrying *m* genes and *n* cell types, we assumed a linear model $\mathbf{y}^k = \mathbf{X} \cdot \boldsymbol{\beta}^k + \mathbf{e}^k$, where $\mathbf{y}^k$ is a $(m \times 1)$ vector representing the simulated expression values of all *m* genes in tissue *k*; the reference data $\mathbf{X}$ is a $(m \times n)$ matrix where $x_{jc}$ denotes the (log-scale) gene-expression value of gene *j* in cell type *c*; and $\mathbf{e}^k = \{e_j^k\}$ is the $(m \times 1)$ vector of normally distributed error terms $e_j^k \sim N(0, \sigma_j^2)$ where $\sigma_j^2 = \sum_{c=1..n} x_{jc} \cdot \gamma_g / n$ is based on a noise factor $\gamma_g$.

The $(n \times 1)$ vector of cell type coefficients, denoted $\boldsymbol{\beta}^k = \{\beta_c^k\}$, is defined as $\beta_c^k = (q_c^k / \sum_{c=1..n} q_c^k) + \varepsilon_c^k$, where $q_c^k$ is the quantity of cell type $c$ in tissue $k$ and $\varepsilon_c^k \sim N(0, q_c^k \cdot \gamma_c / \sum_{c=1..n} q_c^k)$ is based on the noise factor $\gamma_c$. For each sample, cell type quantities were generated as follows: We first selected four groups of high-quantity cell-types and four groups of low-quantity cell types (denoted H and L, respectively). For DMAP, each group consists of a single cell type (altogether 38 groups). For ImmGen, the groups were generated by partitioning of the cell types into 33 groups using hierarchical clustering. This way, two or more reference profiles of the same cell type reside in the same group and do not attain contradicting alterations in cell quantities. The amount of alteration in these cell types is denoted *s* and referred to as the *effect size*. For a cell type $c \in H$, $c \in L$ and $c \notin \{H, L\}$, its quantity was calculated as $q_c^k = (1+s)/n$, $q_c^k = (1-s)/n$ and $q_c^k = 1/n$, respectively. Altogether, the complexity of the problem depends on the number of altered cell-type groups and the effect size parameter.

A single synthetic data collection consisted of 50 pairs of samples, each sample pair consists of a *test sample* $t_k$ that carries a positive effect size (*s*>0) and a control sample $z_k$ without any effect (*s*=0). Each sample pair attained a different effect size, ranging between *s*=0.01 and *s*=0.5. Each collection was generated using the same reference data (ImmGen with *n*=207 in mouse; DMAP with *n*=38 in human) using a fixed noise level ($\gamma_c = \gamma_g = 0.01$).

For each sample pair, we compared the simulated (true) relative cell type quantities versus predicted relative cell type quantities. *True relative cell type quantities* for a given sample pair *k* are defined as $\Delta^k = \boldsymbol{\beta}^{t_k} - \boldsymbol{\beta}^{z_k}$. *Predicted relative cell type quantities* were calculated as follows: using DCQ we first calculated the relative expression data for each sample pair *k* ($\mathbf{y}^{t_k} - \mathbf{y}^{z_k}$ in log-scale) and then applied DCQ on these relative values to attain the $\hat{\Delta}^k$ as output. Using each of the compared deconvolution methods, for each sample pair *k*, deconvolution is first applied on each sample independently to attain predicted fractions ($\hat{\boldsymbol{\beta}}^{t_k}$ or $\hat{\boldsymbol{\beta}}^{z_k}$ values); the predicted relative cell type quantities were then calculated as $\hat{\Delta}^k = \hat{\boldsymbol{\beta}}^{t_k} - \hat{\boldsymbol{\beta}}^{z_k}$.

Here we tested five deconvolution methods. I-NNLS (Abbas, et al., 2009), QProg (Gong, et al., 2011) and DSA (Zhong, et al., 2013) were implemented using the CellMix R implementation (Gaujoux and Seoighe 2013); Cybersort (Newman, et al., 2015) and DCQ (Altboum, et al., 2014) were implemented using the supplemented R script and ComICS (Steuerman, 2016), respectively.

## Supplementary Methods 4: The ImmQuant pipeline

ImmQuant is a software tool for revealing the composition of immune-cell types based on analysis of gene-expression data in complex (heterogeneous) tissues. ImmQuant is operated in three stages (**Figure 1**): first, loading of gene-expression data from heterogeneous samples of interest; secondly, specifying the requested deconvolution algorithm and its parameters, including the desired organism, reference data, log transformation and fold-change calculation; and finally, visualizing the inferred immune cell-type quantities.

**Stage I: Uploading of expression data**. The basic input data constitute a tab-delimited text file, where each row corresponds to a single gene and each column corresponds to a single heterogeneous sample (**Supplementary Figure 1a**). Before proceeding to the next step, it is important to specify the organism and whether the dataset is log-transformed. ImmQuant does not support data normalization, and the input gene-expression file is assumed to be already normalized (see demonstration in **Supplementary Methods 5**).

**Stage II: Choosing deconvolution parameters**. Deconvolution relies on three main parameters: the fold-change calculation, the reference data, and the set of marker genes. ImmQuant offers several pre-compiled settings for each parameter and further allows user-defined configurations (**Supplementary Figure 1b**).

*(i) Choosing the fold-change calculation*. ImmQuant allows two types of fold-change calculations: relatively to the average of all samples and relatively to a selected subset of control samples.

*(ii) Choosing reference data*. ImmQuant contains two optional reference datasets in human (IRIS and DMAP) and one dataset in mouse (ImmGen) (Abbas, et al., 2005; Heng and Painter, 2008; Novershtern, et al., 2011). It is possible, moreover, to add user-defined reference data (that is, a tab-delimited text file containing an expression matrix where rows are genes and columns are cell types), which is essential in the case of non-human/non-mouse investigations.

*(iii) Choosing the set of marker genes.* ImmQuant offers pre-compiled sets of genes for each of the reference datasets. Alternatively it is possible to utilize a user-defined set of markers; such a set is essential in the case of a user-defined reference dataset.

**Stage III: Visualizing the results**. On completion of deconvolution, the inferred relative cell-type quantities can be saved in a tab-delimited text file (where columns are samples and rows are cell types). In addition, the results will be visible in the main window (**Supplementary Figure 1c−e**). The right panel contains the results across all samples (a matrix and a comparative viewer) and the left panel provides the view of the haematopoietic-lineage tree of a single chosen sample (the tab allows switching between

different solutions). It is possible to save all visualizations in a variety of image file formats, including a vector PDF format. Each of these viewers is described below.

*(i) The matrix viewer*. This visualization provides a heat map of cell types (rows) and samples (column), where the red/blue color gradient corresponds to the inferred relative cell-type quantities (**Supplementary Figure 1c**). The visualization allows for zoom-in and zoom-out, sorting of columns and rows, and choosing the range of relative cell-type quantity values.

*(ii) The comparative viewer*. This viewer compares the inferred cell-type quantities of two desired groups of samples (**Supplementary Figure 1d**). The 'Two-sample groups' panel allows the samples in each group to be specified. The output graph is composed of a pair of boxplots for each cell type, one for each desired group of samples.

*(iii) The lineage-tree viewer*. Changes in cell-type quantities involve substantial differentiation from one form of cell type to another. Therefore, to interpret the changes in cell-type quantities it is essential to be able to view the lineage relationships between cell types. To address this, ImmQuant projects the relative cell-type quantities in each sample on top of the haematopoietic-lineage tree (**Supplementary Figure 1e**). In this visualization, each cell type appears as a node and each differentiation pathway as a branch. ImmQuant renders the predicted relative cell-type quantities on the nodes of the tree in colour, where the colour coding is identical to that used in the matrix viewer. The tree provides the inferred relative cell-type quantities for a single sample, and the sample can be selected from a dropdown menu. Once a cell-type lineage tree is loaded, the user can modify the structure of the tree and then utilize this user-defined structure in future analyses.

**Software requirements**. ImmQuant requires pre-installation of Java Runtime Environment (JRE) version 7 or later and R version 3.1.1 or later. During the first execution of ImmQuant, several R packages (ComICS, Glmnet and foreach) are automatically installed.

## Supplementary Methods 5: Real data analysis

DCQ requires a standard pre-processing of the input gene expression data from complex tissues. For example, in the case study of Influenza infection in mice (GSE49934), raw reads of RNA-sequencing were first aligned to the mouse genome (mm9) using the TopHat algorithm and raw expression levels were calculated using the Scripture algorithm. Normalization was then applied using the DESeq method. Using log2-transformed data we normalized each entry by its gene's average and standard deviation across all samples. In the case study of Sjögren's syndrome (GSE40611), Affymetrix GeneChip Human U133 measurements were normalized using the MAS5 algorithm.

# Supplementary Information

## Supplementary Information 1 - Performance evaluation using synthetic data

We have previously developed and confirmed the DCQ algorithm using experiments in mice (Altboum, et al., 2014). Here we used two types of synthetic data collections to test the ability of DCQ to capture alterations in cell type composition: (i) a simulation of mouse samples harbouring a mixture of a large number of cell types from the ImmGen dataset (207 cell types); and (ii) a simulation of human samples consisting of 38 cell types from DMAP (Novershtern, et al., 2011). A main parameter of our synthetic data generation is the 'effect size' - namely, the levels of alterations in cell type quantities between samples. The agreement between simulated and predicted relative cell type quantities was evaluated using the Pearson correlation metric. We compared DCQ to four immune deconvolution approaches: Cibersort (Newman, et al., 2015), DSA (Zhong, et al., 2013), QProg (Gong, et al., 2011) and I-NNLS (Abbas, et al., 2009). A detailed description of data generation and performance evaluation is provided in **Supplementary Methods.**

The analysis revealed the following pattern: with a low number of cell types (~40 cell types in human), Cibersort outperforms DCQ; however, with a large number of cell types (~200 cell types in mouse), DCQ outperforms all other methods (**Supp. Information 1 - Figure 1a,b**). In fact, the alternative methods showed a global decrease in performance as the number of cell types increased, whereas DCQ remained largely unaffected (**Supp. Info 1 - Figure 1c**; see summary in **Supplementary Table 1**). The results remained similar regardless of the effect size. Overall, the analysis highlights the sensitivity of the alternative methods to a large number of cell types. DCQ seems to accurately predict relative cell type quantities across a range of numbers of cell types, both in human and mouse data.

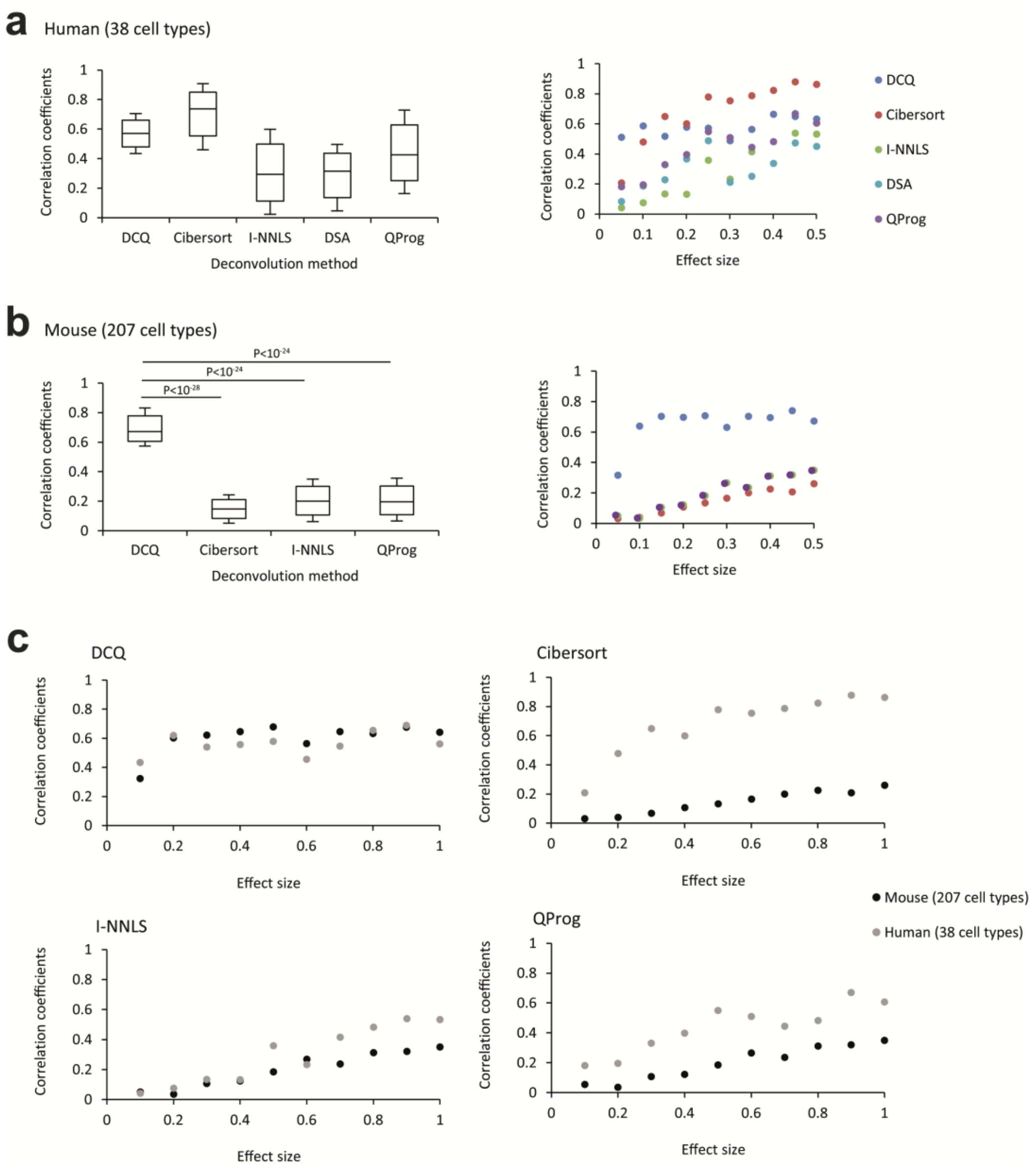


**Supp. Information 1 - Figure 1. Performance analysis using synthetic data.** (**a,b**) Left: shown is a box plot of the correlation coefficients (*y*-axis) that were assessed for different deconvolution methods (*x*-axis) across synthetic tissue pairs. Right: shown is the average correlation coefficients (*y*-axis) that were assessed for different effect sizes (*x*-axis) and various deconvolution methods (color coded). Results are shown for human (**a**) and mouse (**b**) synthetic data collections. The DSA method could not be applied in mouse data due to the large number of cell types. (**c**) Shown is the average correlation coefficients (*y*-axis) that were assessed for different deconvolution methods (sub-panels) and different effect sizes (*x*-axis) using the human (gray) and mouse (black) data collections. Altogether, DCQ outperformed the alternative methods when using the murine dataset. Furthermore, DCQ robustly predicted relative cell-type quantities also in the case of human synthetic data. Cibersort - Newman, et al., 2015; DSA - Zhong, et al., 2013; QProg - Gong, et al., 2011; I-NNLS - Abbas, et al., 2009.

**Supplementary Information 2 - Case studies**

Two case studies were used to exemplify the ImmQuant software. The murine lung response to influenza infection (Altboum, et al., 2014) is a model of dynamic physiological response, whereas the human Sjögren's syndrome (Horvath, et al., 2012) is a model of a sustained pattern. Both datasets are available for download in http://csgi.tau.ac.il/ImmQuant/. A detailed methodological description appears in **Supplementary Methods 5**.

*I. Murine case study—lung tissues following Influenza infection*. The input consisted of 11 expression profiles of the lung complex tissue from C57BL/6J mice at different time points during *in-vivo* infection with the PR8 Influenza virus (GSE15907). ImmQuant was applied on fold changes compared to the non-infected sample using the ImmGen reference data. Notably, the alternative viewers allow addressing two key interpretations. First, the lineage-tree viewer provides information about the control of relative cell-type quantities in each lineage. For example, the screenshot in **Supp. Information 2 - Figure 1a** is focused on 123-h post-infection, and shows an increase in terminally differentiated natural killer T (NKT) cells but not in their intermediate states, and an increase in early rather than in late activated CD8+ effector T cells. More globally, the matrix viewer uncovers the cell-type dynamics during infection. For example, it shows the early decrease in quantities of resident monocytes, accompanied by a late phase increase in inflammatory monocytes, as expected (**Supp. Information 2 - Figure 1b**). Taken together, whereas the matrix viewer allowed characterization of cell-type dynamics, the tree viewer provided details about the control of relative cell-type quantities in different lineages.

*II. Human case study—Sjögren's syndrome*. In this case study, ImmQuant was applied on 16 samples taken from the parotid glands of Sjögren's syndrome patients (a chronic autoimmune disease) using fold changes compared to health individuals (data from (Horvath, et al., 2012)) based on the DMAP reference dataset (Novershtern, et al., 2011). ImmQuant's comparative viewer uncovers a significant disease-related increase in mature and naive B cells, esonophils, HSCs and different granulocyte populations; in addition, we also observe a significant decrease in quantities of erythroid populations (FDR 0.1; **Supp. Information 2 - Figure 2a**). A similar pattern is exemplified using the lineage-tree viewer for a single patient (**Supp. Information 2 - Figure 2b)**. Whereas the changes in B cell quantities have been documented (Ambrosi and Wahren-Herlenius, 2015; Bird, et al., 2015; Cornec, et al., 2012; Hansen, et al., 2007; Mackay, et al., 2007), the role of esonophils, HSCs and neutrophils in Sjögren's syndrome is yet unknown.

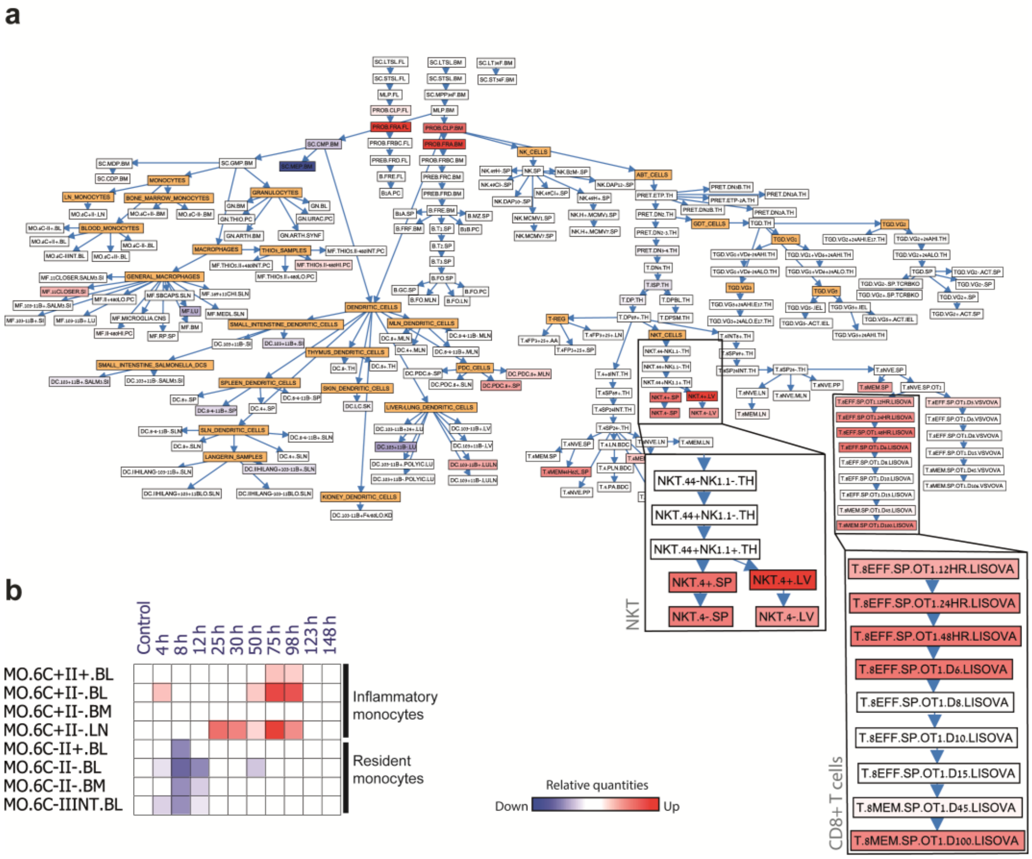


**Supp. Information 2 - Figure 1**. **A lineage-temporal view of inferred cell populations in murine Influenza infection.** (**a**) Control on cell-type quantities in different lineages. Presented is a screenshot of ImmGen's cell-lineage tree, focusing on the complex lung tissue at 123 hours post-infection. Shown is a zoom-in on NKT and CD8+ effector T cell populations (bottom right). (**b**) Cell-type dynamics during the course of infection. Presented is a partial view of the matrix visualization, uncovering the dynamics of specific monocyte subsets (rows) in the course of infection (columns). In both **a** and **b**, results were calculated using the DCQ algorithm with FACS-based marker genes; inferred increase and decrease in cell-type quantities (relatively to time point zero) are indicated in red and blue, respectively.

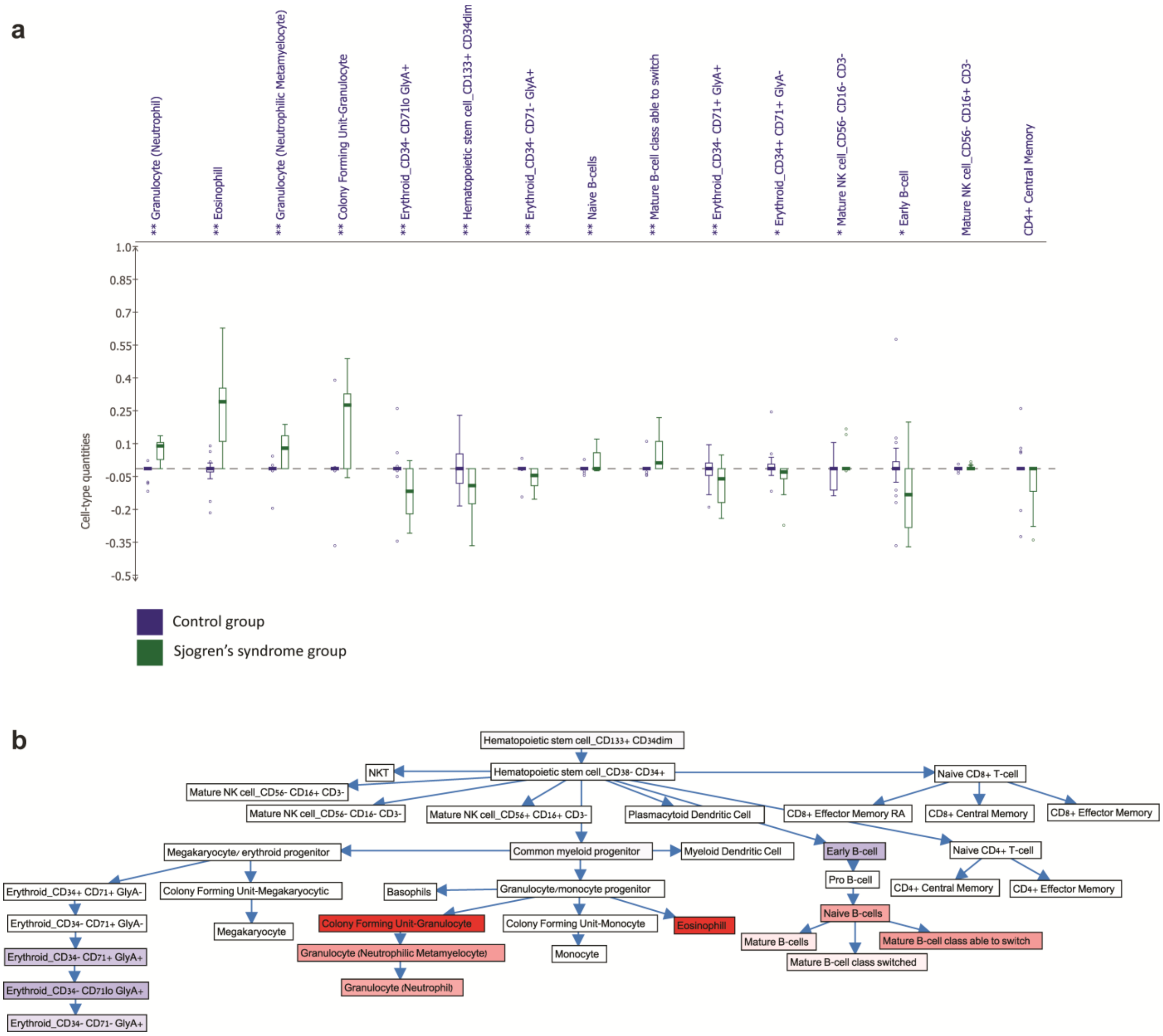


**Supp. Information 2- Figure 2**. **Inferred cell populations in human Sjögren's syndrome.** (**a**) Screenshot of ImmQuant's comparative viewer, highlighting the role of mature and naive B cells, as well as esonophils, granulocyte, HSCs and erythroids (FDR 0.1) in Sjögren's syndrome. (**b**) Screenshot of ImmQuant's lineage-tree view, providing the inferred cell-type quantities in a single patient with Sjögren's syndrome. Blue and red indicate the (relative) low- and high-abundance of cell populations, respectively.

## References

Abbas, A.R., *et al.* (2005) Immune response in silico (IRIS): immune-specific genes identified from a compendium of microarray expression data, *Genes and immunity*, **6**, 319-331.
Abbas, A.R., *et al.* (2009) Deconvolution of blood microarray data identifies cellular activation patterns in systemic lupus erythematosus, *PloS one*, **4**, e6098-e6098.
Altboum, Z., *et al.* (2014) Digital cell quantification identifies global immune cell dynamics during influenza infection, *Molecular systems biology*, **10**, 720-720.
Ambrosi ,A. and Wahren-Herlenius, M. (2015) Update on the immunobiology of Sjögren's syndrome, *Current Opinion in Rheumatology*, **27**, 468-475.
Bird, A.K., Meednu, N. and Anolik, J.H. (2015) New insights into B cell biology in systemic lupus erythematosus and Sjögren's syndrome, *Current Opinion in Rheumatology*, **27**, 461-467.
Cornec, D., *et al.* (2012) B cells in Sjögren's syndrome: from pathophysiology to diagnosis and treatment, *Journal of autoimmunity*, **39**, 161-167.
Gong, T., *et al.* (2011) Optimal deconvolution of transcriptional profiling data using quadratic programming with application to complex clinical blood samples, *PloS one*, **6**, e27156-e27156.
Hansen, A., Lipsky, P.E. and Dörner, T. (2007) B cells in Sjögren's syndrome: indications for disturbed selection and differentiation in ectopic lymphoid tissue, *Arthritis research & therapy*, **9**, 218-218.
Heng, T.S.P. and Painter, M.W. (2008) The Immunological Genome Project: networks of gene expression in immune cells, *Nature immunology*, **9**, 1091-1094.
Horvath, S., *et al* (2012) .Systems analysis of primary Sjögren's syndrome pathogenesis in salivary glands identifies shared pathways in human and a mouse model, *Arthritis research & therapy*, **14**, R238-R238.
Mackay, F., Groom, J.R. and Tangye, S.G. (2007) An important role for B-cell activation factor and B cells in the pathogenesis of Sjögren's syndrome, *Current opinion in rheumatology*, **19**, 406-413.
Newman, A.M., *et al.* (2015) Robust enumeration of cell subsets from tissue expression profiles, *Nature Methods*, **12**, 453-457.
Novershtern, N., *et al.* (2011) Densely interconnected transcriptional circuits control cell states in human hematopoiesis, *Cell*, **144**, 296-309.
Steuerman, Y., Gat-Viks, I (2016) ComICS: Computational Methods for Immune Cell-Type Subsets.
Zhong, Y., *et al.* (2 (013Digital sorting of complex tissues for cell type-specific gene expression profiles, *BMC bioinformatics*, **14**, 89-89.
Zou, H. and Hastie, T. (2005) Regularization and variable selection via the elastic net, *Journal of the Royal Statistical Society: Series B (Statistical Methodology)*, **67**, 301-320.
